# The Associative Changes in Scutellum Nuclear Content and Morphology with Viability Loss of Naturally Aged and Accelerated Aging Wheat (*Triticum aestivum*) Seeds

**DOI:** 10.3389/fpls.2016.01474

**Published:** 2016-09-27

**Authors:** Zaheer Ahmed, Hui Yang, Yong-Bi Fu

**Affiliations:** Plant Gene Resources of Canada, Saskatoon Research and Development Centre, Agriculture and Agri-Food Canada, SaskatoonSK, Canada

**Keywords:** wheat seed aging, DNA alteration, scutellum, aleurone, TUNEL, DAPI

## Abstract

Timely prediction of seed viability loss over long-term storage represents a challenge in management and conservation of *ex situ* plant genetic resources. However, little attention has been paid to study the process of seed deterioration and seed aging signals under storage. An attempt was made here to investigate morphological and molecular changes in the scutellum and aleurone sections of naturally or artificially aged wheat seeds using TUNEL assay and DAPI staining. Twelve wheat genotypes or samples exposed to natural ageing (NA) or accelerated ageing (AA) were assayed and these samples had germination rates ranging from 11 to 93%. The assayed samples showed substantial changes in scutellum, but not aleurone. The nuclei observed in the majority of the scutellum cells of the NA seed samples of lower germination rates were longer in size and less visible, while the scutellum cell morphology or arrangement remained unchanged. In contrast, longer AA treatments resulted in the loss of scutellum cell structure, collapse of cell layers, and disappearance of honey comb arrangements. These nuclei and structural changes were consistent with the DNA assessments of nuclear alternations for the selected wheat samples. Interestingly, the sample seed germination loss was found to be associated with the reductions in the scutellum nuclear content and with the increases in the scutellum nuclei length to width ratio. These findings are significant for understanding the process of wheat seed deterioration and are also useful for searching for sensitive seed aging signals for developing tools to monitor seed viability under storage.

## Introduction

Timely prediction of seed viability loss over the period of seed storage represents a challenge in management and conservation of *ex situ* plant genetic resources (Fu et al., 2015a). Currently, there are 7.4 million accessions of seed germplasm conserved in 1,750 gene banks around the world (FAO, 2010). The long term management of this large volume of valuable genetic resources is a difficult mission. Seeds in long-term storage will eventually lose their viability and assessment of seed deterioration over time is required (Walters et al., 2005; Hay and Probert, 2013; van Treuren et al., 2013). Germination test is the recommended method currently used to assess seed viability (Smith et al., 2003; FAO, 2013). However, this method is not always precise for assessing seed viability (Walters et al., 2005), does not allow for an early prediction of seed viability loss (Roberts, 1973; Pritchard and Dickie, 2003), and cannot reveal underlying mechanisms of seed deterioration (Fu et al., 2015a). Thus, searching and exploring for alternative tools for predicting seed viability loss over time is warranted for better management and conservation of *ex situ* stored seeds (Corbineau, 2012; Nagel et al., 2015). Such exploration, however, requires better understanding of the process and mechanism of seed deterioration and reliable identification of sensitive seed aging signals.

Seed aging or seed deterioration is commonly described as the loss of seed quality or viability over time (Priestley, 1986; Coolbear, 1995). It is a complex biological trait and involves a network of molecular, biochemical, physiological, and metabolic processes (McDonald, 1999; Rajjou et al., 2012). The reported causes for seed deterioration during storage in various plant species include the loss of cellular membrane integrity, weak energy metabolism, production of reactive oxygen species (ROS) and their counter balance, lipid peroxidation, impaired RNA and protein synthesis, enzyme inactivation, and damage to DNA integrity (Priestley, 1986; Vazquez-Ramos et al., 1988; Smith and Berjak, 1995; Walters, 1998; McDonald, 1999; Corbineau et al., 2002; Kibinza et al., 2006). The DNA damage during seed aging can occur either due to an uncontrolled degradation following extensive DNA oxidation in the presence of ROS (Slupphaug et al., 2003) or DNA laddering associated with genetically controlled programmed cell death (PCD; Stein and Hansen, 1999). In spite of these reports, we are still far from understanding of the causes of seed aging and death, particularly under long-term storage in a control environment.

The PCD is an essential process of cereal seed development and occurs in many parts of a developing seed (e.g., see Domínguez and Cejudo, 2014). At the end of the seed development, only some tissues such as embryo axis, scutellum section and aleurone layers remain alive, as these tissues do not undergo PCD during seed development (Domínguez et al., 2012). Also, the scutellum and aleurone layers play an essential role in the germination process by producing the hydrolytic enzymes to mobilize the storage compounds of the starchy endosperm to support early seedling growth (Domínguez and Cejudo, 2014). Malfunctioning in the scutellum and aleurone layer will render a seed unable to germinate. Therefore, the scutellum (SP and SE) and aleurone tissues may provide useful materials suitable for studying aging related deterioration during storage. Any changes leading to its deterioration in these tissues are not directly associated with PCD, but reflect the responses to the later seed aging under various conditions.

We hypothesized that cereal seed aging is associated with irreversible changes in the scutellum and aleurone sections of cereal seeds and conducted a test on the hypothesis in cereal seeds of variable viability using TUNEL assay (Gavrieli et al., 1992) coupled with DAPI staining (Kapuscinski, 1995). For this test, we selected naturally aged (NA) wheat seeds of variable germination rates from the long-term storage at the Plant Gene Resources of Canada (or Canadian national seed bank at Saskatoon) and generated AA wheat seeds under high moisture and high temperature for certain time periods (Delouche and Baskin, 1973). The specific objectives of this study were to (1) assess morphological and molecular changes in the scutellum and aleurone sections of the selected NA or AA wheat seeds using TUNEL assay and DAPI staining and (2) determine the association between the changes in the scutellum and aleurone and seed germination.

## Materials and Methods

### Ethical Standards

The writing process of this manuscript complies with the current laws of Canada.

### Plant Material

This study considered two sets of bread wheat seeds (*Triticum aestivum* L. subsp. *aestivum*): naturally aged germplasm and germplasm exposed to AA (**Table 1**). Naturally aged (NA) germplasm consisted of six accessions that were stored in the 1990s and had a range of germination rates from 11 to 93%. Their selection was made based on a germination profile of 550 accessions generated from the world wheat germplasm collection conserved in Plant Gene Resources of Canada since the 1970s (Fu et al., 2015b). The germination test was conducted following the Association of Official Seed Analysts (1992) guidelines. Briefly, percent germination was measured on three replicates of 100 seeds for each accession. Seeds were placed between the first of three layers of standard germination paper (Anchor Paper, 480 Broadway Street, St. Paul, MN, USA) which had been moistened with 45 ml distilled water. Papers were rolled and kept at 20°C with 8 h light and 16 h darkness in germination cabinets for 7 days before assessing the percentage of normally germinated seedlings.

**Table 1:** The assayed wheat material and their information on storage year, aging treatment, germination rate, extent of microscopy, and DNA assessment of nuclear alternations.

				Microscopy				DNA assessment		
						
Sample	Year	Aging	GR	NS	NSO	PT	LS	DNA(μg)	260/280	260/230
CN2767	1996	NA	92%	8	150	55				
CN2707	1996	NA	93%	10	180	68				
CN44514	1997	NA	55%	10	180	70	M_1_NA	10.1 ± 0.8	1.84	2.01
CN44426	1996	NA	53%	10	180	70	M_2_NA	10.0 ± 1.2	1.84	2.00
CN42511	1995	NA	15%	8	144	31	L_1_NA	4.6 ± 0.5	1.86	2.11
CN43693	1994	NA	11%	9	162	56	L_2_NA	4.5 ± 0.4	1.85	2.09
‘AC Barrie’	2012	For AA	90%	8	140	62	H_1_NA	15.8 ± 3.9	1.81	2.17
‘AC Superb’	2013	For AA	90%	8	150	60	H_2_NA	14.8 ± 2.2	1.89	2.19
‘AC Barrie’	2012	Aged 44h@43	73%	10	180	78				
‘AC Superb’	2013	Aged 48h@43	72%	10	180	68				
‘AC Barrie’	2012	Aged 72h@43	35%	10	180	72	L_1_AA	2.9 ± 0.4	1.84	2.04
‘AC Superb’	2013	Aged 96h@41	20%	10	180	68	L_2_AA	2.8 ± 0.5	1.86	2.04
‘AC Superb’	2015	Freshly harvested	95%				FH	15.2 ± 1.9	1.90	2.20
Total				111	2006	758				


*CN represents the Canadian accession number used in the PGRC collection; Year, the year for the material initially stored; NA, natural ageing in the PGRC collection; GR, germination rate; NS, number of seeds embedded in paraffin for TUNEL and DAPI staining; NSO, number of sections observed under microscope; PT, number of pictures taken; and LS, labeled for the samples used for DNA assessment [HNA, high germination under NA; MNA, medium germination under NA; LNA, low germination under NA; LAA, low germination under accelerated ageing (AA)].*

The germplasm exposed to AA consisted of two accessions (or cultivars; ‘AC Barrie,’ ‘AC Superb’) regenerated in 2012 in a field trial at the Saskatoon Research Centre experimental farm. These recently regenerated accessions were expected to display little impact of NA within 1–2 years of storage. Three random samples of 400 seeds were selected from each accession and two of them were subjected to different AA treatments. The AA was conducted using the inner chamber ‘tray method’ following the overall set-up outlined in Diederichsen and Jones-Flory (2005) and using the time/temperature treatments listed in **Table 1** to achieve germination rates ranging from 20 to 73%.

### Seed Fixation, Processing and Tissue Sectioning

For each aged seed sample, approximately 10 healthy looking wheat seeds were randomly collected and used for paraffin embedding. The whole embedding procedure took up to 8 days. On day 1, a small part of each selected seed was cut off to allow for better infiltration of solution, and the cut seeds were fixed in 2% formaldehyde and 2% glutaraldehyde in 50 mM phosphate buffer (pH 7.5) (PBS) containing 150 mM NaCl overnight at room temperature. On day 2, the fixed material was then dehydrated in a series of ethanol concentrations (50, 70, 80, 90, and 100%) for minimum 5 min and finally 100% ethanol for at least 10 min. The cut tissue in 100% ethanol was left overnight at 4°C. On day 3, the cut tissue was incubated at room temperature for 1 h in 100% ethanol, 4 h in 50% ethanol: 50% Citrisolv (Fisher Scientific cat. no. 22-143-975) solution, and overnight in 100% Citrisolv. On day 4, fresh Citrisolv was replaced. The cut tissue was then submerged in a solution containing 50:50 Citrisolv and Paraplast (SPI Supplies cat. no. 02846-AB) and placed at 60°C in a constant temperature oven (Yamato, DKN 600, Tokyo, Japan). On days 5–7, pure Paraplast was changed each day. Following incubation, the material was embedded in paraffin wax (Parchem, cat. no. 8002-74-2, NY, USA). On day 8, a mold box was placed on a warmer and filled with molten paraffin wax. The tissue was placed in the mold box in a desired orientation with the help of a pre-warmed forceps. The mold was placed in water overnight at room temperature to cool and solidify the paraffin wax. The embedded materials were kept at 4°C until further use.

A razor was used to trim additional wax of the embedded material and to make sure that only single cut tissue was placed in the middle of the small chunk of wax. Sections of 6 μm thickness were cut using Rotary microtome (Leica Rm 2165, Leica Biosystem) in a continuous ribbon form. Each ribbon contained around 6–8 sections. Two ribbons were carefully transferred onto poly-_L_-lysine coated slides and kept at 45°C for a few hours on a slide warmer to make sure the sections wrinkle-free. Separate sectioning was performed for both embryo and endosperm tissues.

### TUNEL Assay, DAPI Staining and Microscopy

DNA strand breaks in the cells were detected using the DeadEnd^TM^ Fluorometric TUNEL System (Promega BioSciences, cat. no. G 3250, CA, USA) according to manufacturer’s instructions. Briefly, the slides with sections were soaked in Citrisolv solution to remove the wax, followed by a series of washes with decreasing concentrations of ethanol (100, 95, 85, 70, and 50%), each for 5 min, and the final wash in 0.85% NaCl and 50 mM PBS. The slides were then fixed in 8% formaldehyde and 8% glutaraldehyde in PBS, followed by proteinase K^+^ (provided with kit) treatment. The TUNEL reaction mixture was added to the sections and the slides were incubated at 37°C for 1 h in a humidified chamber in the dark. The reaction was stopped by placing the slides in 2x saline sodium citrate (SSC) buffer, followed by three washes in PBS. The slides were stained using Vectashield Mounting Medium with DAPI (Vector labs. cat. no. H-1200) staining solution. The stained slides were covered with a cover slip and placed at 4°C in the dark until microscopy was performed.

The embryo and endosperm sections following TUNEL assay and DAPI staining were observed under an Apotome fluorescence microscope (Zeiss AxioImager Z1, Germany). The microscopic imaging was mostly focused on observing variations in scutellum (or SE; SP) and aleurone tissues of embryo and endosperm, respectively. The following parameters were used to scan the fluorescent images of the sections: for green TUNEL signal, excitation wavelength/emission bandpass 470/500–550 nm; for blue DAPI signal, excitation wavelength / emission bandpass 365/420–470 nm. The images were taken using a digital CCD camera (Zeiss AxioCam MRm) mounted on the microscope. The images were processed with the imaging software Zeiss AxioVision 4.8 (Zeiss, Germany).

### DNA Assessment of Nuclear Alterations

To analyze changes in DNA quantity and quality under different aging treatments, eight original samples were chosen to represent various germination rates under different aging treatments (**Table 1**). They are ‘AC Barrie,’ 90% and ‘AC Superb,’ 90% for HNA; CN44426, 55% and CN44514, 53% for MNA; CN42511, 15% and CN43693, 11% for LNA; and ‘AC Barrie’-AA 72h@43°C, 35% and ‘AC Superb’-AA 96h@41°C, 20% for LAA. A sample of FH mature dry seeds of ‘AC Superb’ with 95% germination was selected as positive control. The embryos were collected from the seeds of each selected genotype by cutting the endosperm with a razor blade. The extracted embryos were ground into a fine powder with a mortar and pestle. DNA was extracted from 20 mg of embryo ground powder for each sample using Nucleo Spin^®^ Plant II kit (Macherey-Nagel, Düren, Germany) DNA extraction kit according to manufacturer’s instructions.

The quantity and quality of extracted DNA were first measured by NanoDrop 8000 (Thermo Scientific). To confirm the quality and detect DNA laddering or smearing, approximately 15 μg of extracted DNA from each sample was loaded onto 1.5% agarose gel. The extracted DNAs for the samples of ‘AC Barrie’ (90%) and ‘AC Superb’ (90%) were digested with EcoRI-HF^TM^ (cat. no. R3101L, New England, BioLabs) for 3 h at 37°C and loaded onto 1.5% agarose gel as a negative control. The electrophoresis was carried out in 1x TAE buffer (40 mM Tris-Acetate, 1 mM EDTA) at 100 V and 3 mAh for 3 h. The gels were visualized by staining with 0.5 mg/mL ethidium bromide. The quality of DNA in terms of intactness was also measured by loading 500 ng of DNA from all the samples, including negative and positive controls, onto a 1.5% agarose gel, followed by the same electrophoresis and gel visualization as above. The intensity (pixels) of intact DNA band from each sample was measured by VisionWorks^®^ Life Science Software (Ultra-Violet Products Ltd. Cambridge, UK). These intensities were compared with positive and negative controls and also used to determine the relationship between germination and DNA quality. The DNA assessment was performed with four replications in two independent trials and acquired intensity data are presented as an average.

### Data Analysis

Wheat micrographs were first visually inspected for differences in cell size, shape, arrangements of different cells in a cell layer or arrangements of cell layers in the scutellum and aleurone. As TUNEL assay coupled with DAPI staining was used, differences in nuclei staining (pixels) in these tissues were also evaluated visually. ImageJ software^1^ was used to measure nuclei staining, cell diameter and nuclei size (μm). To determine the differences in the scutellum nuclei staining intensities, approximately 1500 cells in 10–13 micrographs from different seeds of each aging treatment were randomly selected and analyzed without the image background. All the selected images were converted to gray scale and the nuclei were made visible for intensity measurement. The analysis of staining intensity was performed twice for each aging treatment. To measure the scutellum nuclei length to width ratio, approximately 600 cells in 20–25 micrographs from different seeds of each aging treatment were randomly selected and analyzed. Such analysis was repeated once for each aging treatment. For each analysis, the scale was set on ImageJ, the image was zoomed and nuclei length and width were measured manually. The measured length and width values were used to estimate length to width ratios. To assess the differences in the aleurone cell diameters, approximately 1500 cells in 10–13 micrographs from each treatment were randomly selected and the cell diameters in each micrograph were measured using built-in tool in the software. The micrograph selection and cell diameter measurement were done twice for each aging treatment. The means of all the resultant image measurements from two repeated analyses were used to determine and plot the associations of sample germination rates with the measured cell features through a linear regression analysis of SigmaPlot 12.5 statistical and plot software^2^.

## Results

### Changes in Scutellum and Aleurone

**FIGURE 1:**
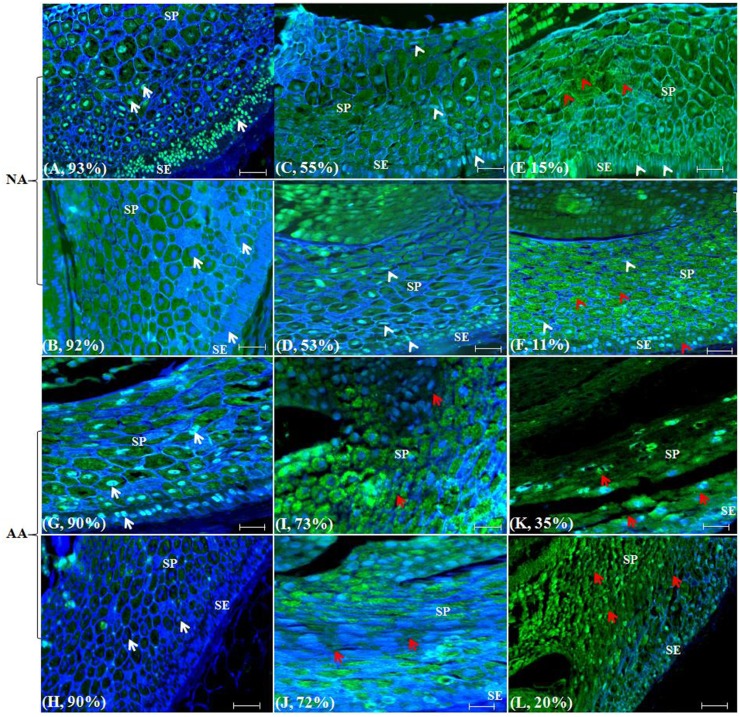
**Wheat scutellum labeled with TUNEL and DAPI to illustrate nuclear and morphological alterations following naturally aged (NA) or accelerated ageing (AA) treatments.** Aged wheat seeds were embedded in paraffin. The scutellum sections were assayed with TUNEL and DAPI staining and visualized under a fluorescence microscope. Each section has a sample label from **(A–L)** and its germination rate, and is highlighted with narrows. Specifically, **(A)**, CN2707-1996; **(B)**, CN2767-1996; **(C)**, CN44514-1997; **(D)**, CN44426-1996; **(E)**, CN42511-1995; **(F)**, CN43693-1994; **(G)**, AC Barrie-2012; **(H)**, AC Superb-2013; **(I)**, AC Barrie-AA 44h@43°C; **(J)**, AC Superb-AA 48h@43°C; **(K)**, AC Barrie-AA 72h@43°C; and **(L)**, AC Superb-AA 96 h @41°C. White arrows indicated normal well stained round nuclei; white arrow heads, longer nuclei; red arrow heads, cell or cell layers devoid of nuclei; red arrow, cells or cell layers with altered morphology; SE, scutellum epthelium; and SP, scutellum paranchyma. Scale bar represents 50 μm.

We generated a total of 2,006 sections from 111 wheat seeds representing NA or AA aging treatments and different levels of germination and produced 758 micrographs (**Table 1**). Examining these micrographs revealed considerable changes in scutellum, but not aleurone, of naturally aged and AA wheat seeds. The following patterns of changes in scutellum were obtained through a summary of detailed inspection on all related micrographs for each aging treatment and illustrated in **Figure 1**.

Scutellum changes were dramatic in naturally aged wheat seeds (**Figures 1A–F**). The genotypes CN2707 and CN2767 with 92–93% germination (**Table 1**) were used as negative controls. For these two genotypes, the scutellum (SE and SP) possessed clearly visible well DAPI stained round nuclei in the majority of the cells (**Figures 1A,B**, white arrows). The two other NA genotypes CN44514 and CN44426 with 53–55% germination showed that the nuclei in the scutellum (SE and SP) cells were longer in size, less stained with DAPI and thus less visible (**Figures 1C,D**, white arrow heads) when compared to those of negative controls. For the NA genotypes CN42511 and CN43693 with 11–15% germination, the nuclei in the majority of the scutellum (SE and SP) cells were long and condensed, merely stained by DAPI and hence displayed minimum visibility (**Figures 1E,F**, white arrow heads). Due to less visibility, the majority of the cells or cell layers in the scutellum (SE and SP) seemed to be devoid of nuclei (**Figures 1E,F**, red arrow heads). Note that the patterns of longer and condensed nuclei were also visible in some cells of the scutellum (SE and SP) in negative controls, but the extent of this scutellum cell change was greatly increased in the NA genotypes with lower germination rates. Interestingly, the cell morphology or cell layers arrangements within the scutellum remain largely unchanged. The normal honey combed arrangement was retained in SP regardless of germination level (**Figures 1A–F**).

Scutellum changes also occurred in AA seeds (**Figures 1G–L**). The genotypes ‘AC Barrie’ and ‘AC Superb’ with 90% germination (**Table 1**) were used as negative controls for AA seeds. For these two genotypes, the scutellum (SE and SP) possessed clearly visible well DAPI stained round nuclei (**Figures 1G,H**, white arrows) similar to those observed in NA negative controls. The two AA seed samples (‘AC Barrie,’ 44h@43°C and ‘AC Superb,’ 48h@43°C) with 72–73% germination displayed considerable changes in the scutellum (SE and SP) cells and nuclei morphologies when compared to the negative controls. In both AA genotypes, nuclei became longer in size, and cells and cell layers in the scutellum, particularly SP, started to lose their integrity. The deformation of proper honey comb arrangements in SP started to appear (**Figures 1I,J**, red arrows). Also, the extent of deformation was appeared to be larger in the AA genotype ‘AC Superb’ than ‘AC Barrie,’ probably due to the fact that ‘AC Superb’ was exposed to the slightly longer AA treatment than ‘AC Barrie’ to achieve roughly the same level of germination (**Table 1**; **Figures 1I,J**). With longer AA treatments, the two AA seed samples (‘AC Barrie,’ 72h@43°C and ‘AC Superb,’ 96h@41°C) displayed much reduced germination (35% for ‘AC Barrie’ and 20% for ‘AC Superb’) (**Table 1**). The exposure to longer AA treatment resulted in the loss of scutellum (SE and SP) cell structure, collapse of cell layers, and disappearance of honey comb arrangements. Such structural changes made the nuclei and other cell contents not observable in most of the SE and SP cells and cell layers in both AA seed samples (**Figures 1K,L**, red arrows). The extent of cellular damage appeared to be larger in AA samples of ‘AC Superb’ than ‘AC Barrie’ and consistent with the lower germination level observed for the AA sample of ‘AC Barrie.’

**FIGURE 2:**
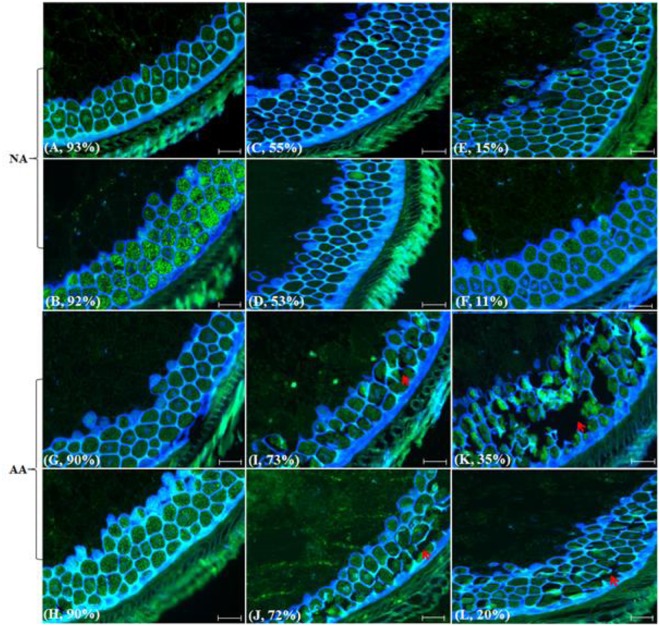
**Wheat aleurone labeled with TUNEL and DAPI to illustrate nuclear and morphological alterations following NA or AA treatments.** Aged wheat seeds were embedded in paraffin. The endosperm sections containing aleurone cell layers were assayed with TUNEL and DAPI staining, and visualized under a fluorescence microscope. Each section has a sample label from **(A–L)** and its germination rate, and/or is highlighted with narrows. Specifically, **(A)**, CN2707-1996; **(B)**, CN2767-1996; **(C)**, CN44514-1997; **(D)**, CN44426-1996; **(E)**, CN42511-1995; **(F)**, CN43693-1994; **(G)**, AC Barrie-2012; **(H)**, AC Superb-2013; **(I)**, AC Barrie-AA 44h@43°C; **(J)**, AC Superb-AA 48h@43°C; **(K)**, AC Barrie-AA 72h@43°C; and **(L)**, AC Superb-AA 96 h @41°C. Red arrows indicate cells or cell layers with altered morphology. Scale bar represents 50 μm.

Interestingly, there were no significant changes detected in the layer cells, morphology and nuclear content of aleurone among different NA and AA genotypes or samples (**Figure 2**). However, longer AA treatments seemed to result in the loss of cellular integrity and the broken cell layers (**Figures 2I,J**) and such damage was intensified with an increased AA duration (**Figures 2K,L**). Also, the damage in aleurone from AA treatment was dependent on genotype background. For example, the AA sample (‘AC Barrie’, 72h@43°C) displayed more cell disruption than the AA sample (‘AC Superb,’ 96h@41°C), as the latter possessed more intact cells and cell layers. In contrast to the NA genotypes with variable germination rates, such damage in the aleurone cells morphology was not visible (**Figures 2C–F**).

### DNA Assessment of Nuclear Alterations

To validate the observed reduction in nuclear content as described above, a DNA assessment was made on eight different genotypes representing different aging treatments and germination rates (see Materials and Methods) and FH genotype as a positive control (**Table 1**). The genotypes H_1_NA, H_2_NA and FH with 90–95% or higher germination possessed the highest (±15 μg) of total DNA among the assayed genotypes. The NA genotypes M_1_NA and M_2_NA exhibited around 50% loss in germination and yielded approximately 10 μg of total DNA. The NA genotypes L_1_NA and L_2_NA displayed 11–15% germination and revealed around 5 μg of total DNA. More interestingly, the AA genotypes L_1_AA and L_2_AA under the strong AA treatments had 20–35% germination rates and yielded only around 3 μg of total DNA. Such DNA contents from the AA seeds were lower than the NA genotypes L_1_NA and L_2_NA with lower germination rates.

**FIGURE 3:**
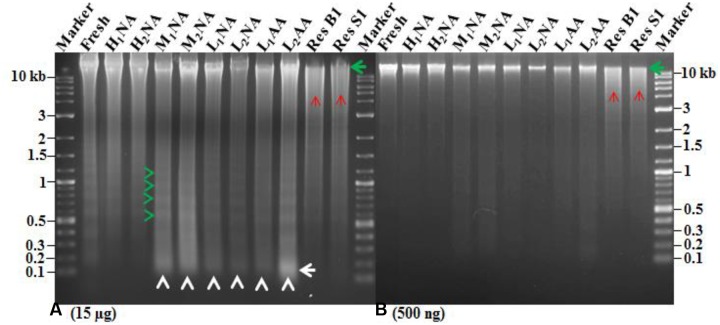
**Agarose gels to illustrate DNA alterations in extracted DNAs of nine aging wheat samples with various germination rates.** **(A)** and **(B)** Show an equal amount of extracted DNA per sample (**A**: 15 μg and **B**: 500 ng). The sample labels are listed in **Table 1**. Res B1 and S1 represent the DNA samples for AC Barrie and AC Superb digested by *Eco*RI enzyme for 3 h at 37°C, respectively. Green arrows indicate intact DNA; red arrows, DNA smearing in *Eco*RI digested DNA; green arrow head, DNA laddering; white arrow heads, DNA smearing; and white arrow, increased DNA smearing in AA wheat genotype.

DNA quality was also assessed by loading 15 μg of total DNA onto 1.5% agarose gel (**Figure 3A**). The NA genotypes H_1_NA, H_2_NA, and FH exhibited more intact DNA and less DNA laddering (**Figure 3A**, green arrow) compared to other assayed genotypes. For the six genotypes (M_I_NA, M_2_NA, L_1_NA, L_2_NA, L_1_AA, and L_2_AA), less intact DNA was observed with more DNA laddering in multiple of 180–200 bp or DNA smearing (**Figure 3A**, green and white arrow heads), respectively. The highest DNA smearing as visualized by intensity was observed in the genotype L_2_AA (**Figure 3A**, white arrow). We also employed two negative DNA controls (i.e., DNA extracted from the genotypes ‘AC Barrie’ and ‘AC Superb’) with digestion by *Eco*RI enzyme. Our controls (**Figure 3A**, Res B1 and Res S1) showed that the intensity of intact DNA band was considerably reduced and more DNA smearing was observed (**Figure 3A**, red arrows). This DNA smearing was more resembling to DNA smearing observed in L_1_AA and L_2_AA (**Figure 3A**, white arrow heads). However, DNA smearing was considerably higher in the negative controls compared to the AA genotypes. One interesting observation was the different position of intact bands for these assayed genotypes.

To provide further confirmation on the quality, the intensity of intact band from all these genotypes were measured by loading 500 ng of total DNA onto 1.5% agarose gel (**Figure 3B**). The highest intact band intensity was observed in the NA genotypes H_1_NA, H_2_NA, and FH (**Figure 3B**, green arrow). The intensity of intact band decreased with reduced germination rate. The intensity of intact band further decreased in the AA genotypes L_1_AA and L_2_AA (**Figure 3B**, green arrow), followed by the negative controls with *Eco*RI digestion (**Figure 3B**, green arrow). We detected a unique pattern in the reduction of intact band intensity as: H_1_NA, H_2_NA, FH > M_1_NA, M_2_NA > L_1_NA, L_2_NA > L_1_AA, L_2_AA > Res B1, Res S1. This pattern confirmed that DNA intactness is associated with seed germination.

### Associations of Germination with Scutellum and Aleurone Changes

**FIGURE 4:**
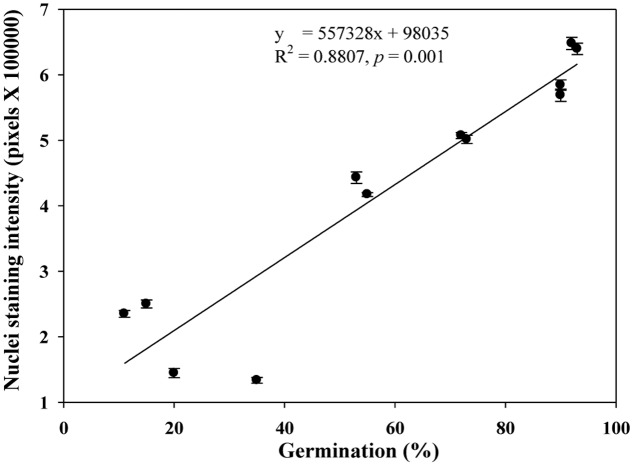
**A linear relationship observed between estimates of nuclei staining intensity and germination rate in naturally aged and AA wheat samples.** Error bar indicates standard error.

**FIGURE 5:**
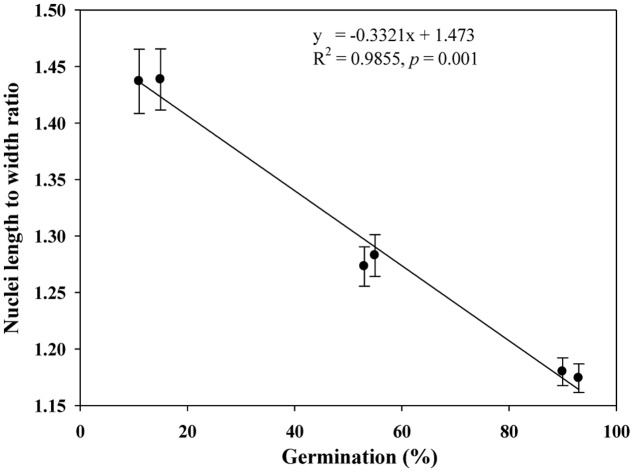
**A linear relationship observed between estimates of nuclei length to width ratio and germination rate in naturally aged wheat samples.** Error bar indicates standard error.

**FIGURE 6:**
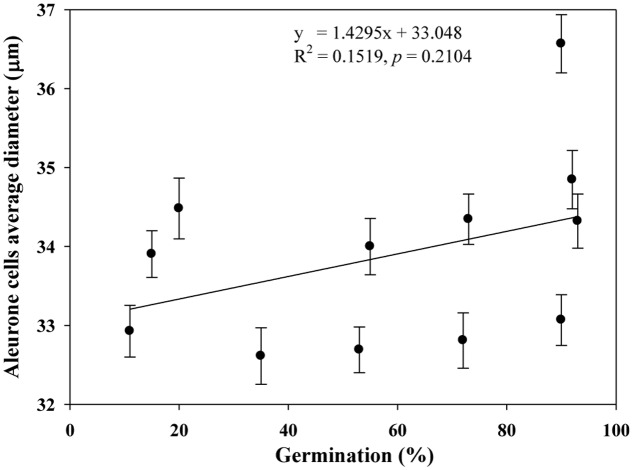
**No significant association observed between estimates of germination rate and aleurone cells average diameter in naturally aged and AA wheat samples.** Error bar indicates standard error.

We quantified the scutellum changes by measuring scutellum and aleurone nuclei intensities, and determined the association between sample germination and nuclei intensity. It was found that an increase in nuclei staining intensity was positively associated with higher germination in NA and AA wheat seed samples (**Figure 4**). Similarly, we measured the scutellum (SE and SP) nuclei length and width and estimated the length to width ratios for the NA wheat seed samples. It was found that an increase in nuclei length to width ratios was negatively associated with increased germination for the NA wheat seed samples (**Figure 5**). However, the ratio measurement was not made for the AA seed samples, as longer AA treatments resulted in the loss of cellular contents and consequently fewer cells with nuclei were available for measurement. Also, we measured the changes in diameter of aleurone cells for both NA and AA wheat seed samples and found a non-significant association between the changes in aleurone cell diameter and germination rate (**Figure 6**).

**FIGURE 7:**
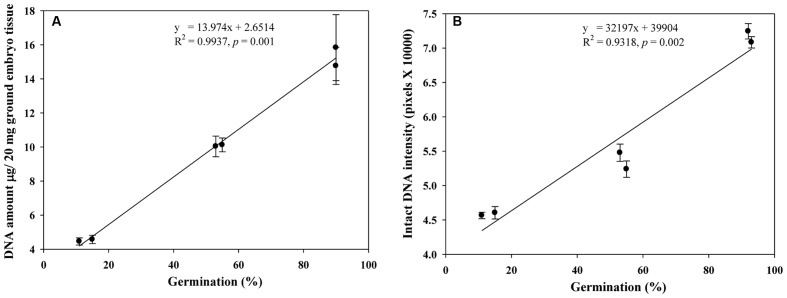
**Linear relationships observed of germination rates with DNA quantities **(A)** and intact DNA intensities **(B)** in naturally aged wheat samples.** Error bar indicates standard error.

We also assessed DNA quantity (μg/20 mg of ground embryo tissue) and quality (intensity of intact DNA) for the selected NA genotypes of variable germination rates (**Table 1**) and found that both DNA quantity and quality were positively associated with increased germination (**Figures 7A,B**). Such association was not assessed for the AA wheat seed samples due to the small sample size (only from two samples L_1_AA and L_2_AA under one AA treatment).

## Discussion

Our study represents the first attempt with a large experimental effort using TUNEL assay and DAPI staining to assess the associative changes in the structures of scutellum and aleurone of aged wheat seeds under NA and AA treatments. The assayed samples showed substantial changes in scutellum, but not aleurone (**Figures 1** and **2**). The nuclei in the scutellum cells of the NA seed samples became longer in size and less visible, while the scutellum cell morphology remained unchanged. In contrast, longer AA treatments resulted in the loss of scutellum cell structure, collapse of cell layers, and disappearance of honey comb arrangements (**Figures 1** and **2**). Interestingly, the sample seed germination loss was found to be associated with the reductions in the scutellum nuclear content and with the increases in the scutellum nuclei length to width ratio (**Figures 4** and **5**). These findings are significant for understanding seed aging process at cellular and molecular levels and useful for searching for sensitive seed aging signals for developing tools to monitor seed deterioration under long-term storage.

The finding of the associative changes in the assayed tissues with seed viability confirmed our scientific reasoning or hypothesis that cereal seed aging is associated with irreversible changes in the scutellum and aleurone sections of cereal seeds. Our reasoning was largely based on the role of the scutellum and aleurone layers in the germination process by producing the hydrolytic enzymes to mobilize the storage compounds of the starchy endosperm to support early seedling growth. In this process, synthesis and secretion of hydrolytic enzymes initially occur in the SE cells (Domínguez and Cejudo, 2014). Then, the aleurone cells, which surround the starchy endosperm, become activated and secrete a large amount of hydrolases (Fincher, 1989). Such process is regulated by gibberellins synthesized in the scutellum and then released into the starchy endosperm (Appleford and Lenton, 1997). Thus, any malfunctioning in the scutellum and aleurone sections will affect seed germination. The unique feature of these tissues without the direct influence of PCD in dry seeds also allowed for the better assessment of the associative changes in these tissues with seed aging, as demonstrated in this study.

The TUNEL assay and DAPI staining are nuclei specific procedures, but they also allow for a direct assessment on cell morphologies in the scutellum and aleurone sections of single seeds. The observed changes in the scutellum during aging gained further support from the population-level analysis of DNA quantity and quality in aged seeds. Based on previous reports (e.g., Rajjou et al., 2012), a dry seed upon rehydration under favorable conditions starts to repair DNA damages to initiate the germination process, but seed will not germinate if the damages are beyond the repair or repair mechanism does not work properly. The nuclear alterations were also considered as key features in soybean (*Glycine max*) and safflower (*Carthamus tinctorius*) seed ageing (Shatters et al., 1995; Vijay et al., 2009). Also, the contrasting observations of DNA laddering in NA or AA seeds may reflect different modes of DNA damage under two aging treatments. The observed DNA laddering during NA may have occurred due to slow enzymatic degradation over time, while the DNA smearing during AA may have occurred due to exposure to temperature and high moisture rather than the enzymatic degradation. These DNA alterations under aging treatments can cause extensive rearrangements in DNA and reduce DNA amounts as observed for samples of low germination rates (**Table 1**). Therefore, assessing nuclear alterations under NA or AA treatments could also provide useful information on wheat seed aging.

To help understand the associative changes observed in scutellum and aleurone layers, extra attempt was made to measure the ROS level and the activities of caspases-like and four antioxidant enzymes (ascorbate peroxidase, catalase, glutathione reductase, and superoxide dismutase) in the assayed materials, following the methods of Maraschin et al. (2005) and Hu et al. (2012). The effort revealed a trend for increased ROS level and increased activities of these antioxidant and caspases-like enzymes in the NA and AA wheat seeds of reduced germination rate (Zaheer Ahmed, unpublished results). This result, along with those from TUNEL assay and DNA laddering, suggested that PCD would have occurred in those aged wheat seeds and ROS production may have involved with the seed deterioration. Similar reports on ROS production and PCD have been found in deteriorated seeds of other species (Kranner et al., 2006, 2010; Egorova et al., 2010; El-Maarouf-Bouteau et al., 2011; Hu et al., 2012). Thus, it is possible that both ROS and PCD were associated with the changes detected in the scutellum and aleurone layers of those assayed seeds. However, our original research was not specifically designed for PCD detection and the extra analysis was preliminary. More systematic investigation is needed to confirm the PCD occurrence in aged wheat seeds, interpret the associative changes observed in scutellum and aleurone layers, and understand the causes of seed aging. Also, more research should be directed to determine whether similar changes would occur in the aging of oily seeds such as flax or canola seeds. Moreover, our analysis of the associative changes would be more informative if we assayed more seed samples or seeds from other cereal crops. Such assays would help to determine the generality of the associative changes in the scutellum and aleurone sections of cereal seeds.

The finding of the associative changes in these two tissues is encouraging for the argument we presented for searching for useful seed aging signals to develop sensitive tools for monitoring seed viability loss over long-term storage (Fu et al., 2015a), as it helps to demonstrate the search for sensitive aging signals can be fruitful. The associative changes found in scutellum were not directly influenced by PCD, but reflected the aging responses under two aging treatments. However, how to utilize the detected association for prediction of seed viability loss remain to be determined, as such detection is relied on advanced equipment and labor-intensive. Also, the detection is expensive and less applicable to screen the large number of seed samples conserved in the genebanks. Realizing these caveats should enhance our search for alternative seed aging signals.

## Author Contributions

ZA performed the experiment including TUNNEL assay, DAPI staining, microscopy, DNA assessment and data analysis, and wrote and edited the paper. HY performed paraffin embedding and sectioning; and edited the paper. YBF conceived and designed the research, and wrote and edited the paper.

## Conflict of Interest Statement

The authors declare that the research was conducted in the absence of any commercial or financial relationships that could be construed as a potential conflict of interest.

## Footnotes

1 imagej.nih.gov

2 http://www.sigmaplot.com

## Abbreviations

AA: accelerated aging
DAPI: 4′,6-diamidino-2-phenylindole
FH: freshly harvested
HNA: high germination under natural aging
LAA: low germination under accelerated aging
LNA: low germination under natural aging
MNA: medium germination under natural aging
NA: natural aging
SE: scutellum epithelium
SP: scutellum parenchyma
TUNEL: terminal deoxynucleotidyl transferase mediated dUTP nick end labeling
